# 12-Month prevalence of coronary heart disease in Germany

**DOI:** 10.17886/RKI-GBE-2017-018

**Published:** 2017-03-15

**Authors:** Markus A. Busch, Ronny Kuhnert

**Affiliations:** Robert Koch Institute, Department for Epidemiology and Health Monitoring, Berlin, Germany

**Keywords:** MYOCARDIAL INFARCTION, CORONARY HEART DISEASE, DISEASE OUTCOMES, GENERAL POPULATION, GERMANY

## Abstract

The results of the GEDA 2014/2015-EHIS study demonstrate that during the last 12 months, 3.7% of women and 6.0% of men in Germany had coronary heart disease (CHD – defined as myocardial infarction, chronic consequences of myocardial infarction or angina pectoris). The 12-month prevalence of CHD in men and women under 45 years of age is well below 1.0%; however, the prevalence rises rapidly and disproportionately up to 16.0% among women and 24.1% among men aged 75 years and over. Women with a low level of education have a considerably higher prevalence of CHD (7.3%) compared to those with a high level of education (1.2%). Men show fewer education-related differences (6.5% versus 5.2%). As the indicators analysed here were first deployed as part of the 2014/2015 European Health Interview Survey (EHIS), no comparative data is available.

## Introduction

Coronary heart disease or ischemic heart disease (CHD) is a chronic disease of the heart in which atherosclerosis leads to progressive narrowing of the coronary arteries. This results in reduced supply of blood to the heart muscle [1]. Angina pectoris (chest tightness) is a frequent symptom in an advanced stage and is characterised by sudden pain behind the sternum. Common complications of CHD include myocardial infarction (heart attack), heart failure and arrhythmia, all of which are associated with a high mortality rate and significant deterioration to health.

CHD is the leading cause of death in Germany and throughout the world [1, 2]; it also results in high costs to health care systems [3, 4]. The rate of new cases (incidence rate) and the mortality of CHD have been falling continuously in Germany for several decades [1]. This is most likely due to improvements in health-related behaviour (people refraining from smoking, increasing their level of physical activity and eating a balanced diet), in cardiometabolic risk profiles (high blood pressure, blood sugar levels, lipid metabolism) [5] and in the care provided to people with acute myocardial infarction [6]. Changes to the mortality rate or the incidence rate also affect the frequency of coronary heart disease found in the population (the prevalence of CHD). In addition, changes to the structure of the population, such as an increased proportion of older people due to demographic change, can also cause a numerical increase in CHD [1]. Therefore, it is essential that the age and gender-specific prevalence be regularly assessed in order to promptly identify positive and negative trends in CHD in the population, and to determine the potential for prevention and the population’s need for medical care.

**GEDA 2014/2015-EHIS**

**Table:** 

**Data holder:**	Robert Koch Institute
**Aims:**	to provide reliable information about the population’s health status, health-related behaviour and health care in Germany, with the possibility of a European comparison
**Method:**	questionnaires completed on paper or online
**Population:**	people aged 18 years and above with permanent residency in Germany
**Sampling:**	registry office sample; randomly selected individuals from 301 communities in Germany were invited to participate
**Participants:**	24,016 people (10,872 men; 13,144 women)
**Response rate:**	26.9%
**Study period:**	November 2014 – July 2015
**Data protection:**	all participants were informed about the study’s aims and content and about data protection, and provided their informed consent

More information is available at

www.geda-studie.de

Two indicators have been introduced into European health monitoring that can be used to measure the frequency of myocardial infarction and its chronic consequences, as well as chronic CHD or angina pectoris, in EU countries over time [7]. These indicators were measured in Germany for the first time as part of the German Health Update (GEDA) 2014/2015 study, which was conducted by the Robert Koch Institute within the framework of the European Health Interview Survey (EHIS) 2014/2015 [8]. More detailed information about health monitoring and health indicators in Europe can be found in a Focus article published in this issue [9].

## Indicator

In order to collect data on numerous disorders and conditions, the GEDA 2014/2015-EHIS study posed the question ‘During the past 12 months, have you had any of the following diseases or conditions?’ This question was followed by a list of illnesses; this enabled information to be collected about ‘myocardial infarction (heart attack)’, ‘chronic consequences of myocardial infarction’ and ‘coronary heart disease or angina pectoris’ in the past 12 months. Participants were able to complete the GEDA 2014/2015-EHIS questionnaire on paper or online. The two indicators from European health monitoring used in the analyses presented here were defined as 1) the presence of a myocardial infarction or chronic consequences of a myocardial infarction in the last 12 months, and 2) the presence of coronary heart disease/angina pectoris in the last 12 months [7]. Since myocardial infarction and chronic CHD/angina pectoris are ultimately manifestations of the same disease of the coronary arteries, and because there are major overlaps between the two health problems, the two EHIS indicators are summarised in the following as CHD. Defining CHD as either myocardial infarction or other forms of coronary heart disease is established practice in international health reporting [1, 3, 10-12].

The following analyses are based on data from 22,639 respondents aged 18 years and above after 1,377 respondents (5.7% of the total sample) were excluded from the analyses because valid information covering their 12-month prevalence of myocardial infarction or chronic consequences of myocardial infarction and coronary heart disease/angina pectoris was missing. All analyses were carried out using a weighting factor that corrected for deviations in the sample from the population structure (as of 31 December 2014) in terms of gender, age, municipality type and level of education. The International Standard Classification of Education (ISCED) was used to ensure that the data gathered from the participants on education was comparable [13]. A detailed description of the methodology applied in GEDA 2014/15-EHIS can be found in the article German Health Update – new data for Germany and Europe [8], which is published in this issue.

## Results and discussion

Over the last 12 months, a total of 3.7% of women and 6.0% of men suffered from coronary heart disease (CHD; defined as a myocardial infarction, chronic consequences of myocardial infarction, or angina pectoris – see Table 1). The 12-month prevalence of coronary heart disease in people under 45 years is well below than 1%; however, it increases disproportionately to 16.0% in women and 24.1% in men aged over 75. Women with a low level of education report considerably more often that they had CHD in the last 12 months (7.3%) compared to women with a high level of education (1.2%). Men show fewer education-related differences (6.5% versus 5.2%).

The indicators described here stem from European health monitoring [1, 11, 12, 14]. The results for the aggregated CHD indicator confirm the known correlation between CHD and increasing age, male gender and lower socioeconomic status among adults in Germany [1, 14]. A comparison to the rest of Europe demonstrates a very low level of variability in the 12-month prevalence of myocardial infarction and its chronic consequences or coronary heart disease between the EU member states [9].

A direct comparison of prevalences for the new EHIS indicators with other epidemiological data is only possible to a limited extent. There are a number of methodological reasons for this. On the one hand, the EHIS study measured myocardial infarction together with chronic consequences of myocardial infarction. Consequently, the EHIS study for the first time uses a mixed indicator that is more strongly influenced by the participants’ subjective perceptions, which are also likely to be affected by their education, gender and other factors. On the other hand, the EHIS study did not ask directly whether an illness had been diagnosed by a physician; doing so is standard practice in international health surveys [11, 12, 14]. Therefore, it is highly likely that the participants’ subjective perceptions and patterns of interpretation affected the level of prevalence identified by the EHIS study more strongly than is usually the case.

In addition, epidemiological studies and health reporting [1, 11, 12, 14] normally study lifetime prevalence; in contrast, EHIS for the first time measured the 12-month prevalence and applied it to comparisons between the countries in Europe. The last studies that estimated lifetime prevalence of CHD in Germany were the GEDA studies 2009-2012: they estimated lifetime prevalence to be 6.6% in women and 9.6% in men [1]. Moreover, there was no evidence of a change in prevalence over the preceding 12 years or of major differences with other countries [1, 14]. The fact that the prevalence among men and women described here is considerably lower than the lifetime prevalence mentioned above can be explained by the short 12-month reference period used in the EHIS study. In addition, as the 12-month prevalence rates are significantly lower, despite the inclusion of chronic consequences of myocardial infarction, this could indicate that a high proportion of people who survive a myocardial infarction do not experience chronic secondary symptoms. As pointed out above, however, the validity of the mixed indicator is difficult to judge due to the inclusion of perceived chronic consequences.

The questionnaire used in the EHIS study was developed through an extensive process of consultation undertaken between the 28 European countries [7-9]. With regard to the questions posed about chronic diseases, the decision was taken to record perceived illnesses instead of the usual focus on physician-diagnosed diseases. Moreover, in the case of myocardial infarction and stroke, chronic consequences of the diseases were also measured. This decision was taken because the results it would provide were viewed as of higher public health relevance than would have otherwise been the case. This decision was also based on the assumption that results would be less strongly influenced by regional care differences [7, 9, 15]. Furthermore, the 12-month prevalence was measured in order to demonstrate the current extent of the disease and to reduce recall bias in data collection [7, 9, 15]. The methodological difficulties described above and the lack of comparability with other data sources suggest that it is unlikely that the EHIS indicators measuring myocardial infarction und coronary heart disease, as well as the indicators for stroke (compare Fact sheet on stroke in this issue [15]), will be applied in other studies.

Despite these limitations, the new EHIS indicators are still of value for harmonised, indicator-supported health monitoring in the EU. Moreover, they can help to identify regional inequalities, positive and negative trends and the potential for disease prevention in European populations as well as to demonstrate the areas of public health in which action needs to be taken.

## Key statements

- In the last 12 months, 3.7% of women and 6.0 % of men in Germany suffered from coronary heart disease (CHD – defined as myocardial infarction, chronic consequences of myocardial infarction or angina pectoris).
- The 12-month prevalence of CHD rises disproportionately with age to approximately 16% among women and 24% among men aged 75 years and above.
- Women with a low level of education reported considerably more often that they had CHD compared to women with a high level of education. Men show fewer education-related differences.

## Figures and Tables

**Table 1:** 12-month prevalence of coronary heart disease (myocardial infarction, chronic consequences of myocardial infarction or chronic coronary heart disease/angina pectoris) by gender, age and educational status (n=22,639) Source: GEDA 2014/2015-EHIS

Women	%	(95%-CI)	Men	%	(95%-CI)
**Women total**	**3.7**	**(3.3-4.2)**	**Men total**	**6.0**	**(5.5-6.5)**
**Age**			**Age**		
18 – 44 years	0.2	(0.1-0.4)	18 – 44 years	0.4	(0.2-0.7)
45 – 54 years	0.9	(0.6-1.5)	45 – 54 years	3.4	(2.5-4.6)
55 – 64 years	3.4	(2.5-4.6)	55 – 64 years	7.7	(6.2-9.4)
65 – 74 years	7.1	(5.6-8.8)	65 – 74 years	13.0	(11.2-15.0)
≥75 years	16.0	(13.3-19.2)	≥75 years	24.1	(21.1-27.4)
**Education**			**Education**		
Low	**7.3**	(6.1-8.7)	Low	**6.5**	(5.3-8.0)
Medium	**3.1**	(2.5-3.7)	Medium	**3.1**	(2.5-3.7)
High	1.2	(0.8-1.8)	High	1.2	(0.8-1.8)
**Total (women and men)**	**4.8**	**(4.5-5.2)**	**Total (women and men)**	**4.8**	**(4.5-5.2)**

CI=Confidence interval
